# 772 Bismuth Tribromophenate Gauze is Superior to Silver Sulfadiazine Cream for Mixed-depth Scald Burns in Children

**DOI:** 10.1093/jbcr/irad045.247

**Published:** 2023-05-15

**Authors:** Dominic Alessio-Bilowus, Nishant Kumar, Ryan Nolan, Justin Klein, Elika Ridelman, Christina Shanti

**Affiliations:** Department of Surgery, Division of Pediatric Surgery, Children's Hospital of Michigan/Wayne State University School of Medicine, Detroit, Michigan; Department of Surgery, Division of Pediatric Surgery, Children's Hospital of Michigan/Wayne State University School of Medicine, Detroit, Michigan; Department of Surgery, Division of Pediatric Surgery, Children's Hospital of Michigan/Wayne State University School of Medicine, Detroit, Michigan; Department of Surgery, Division of Pediatric Surgery, Children's Hospital of Michigan/Wayne State University School of Medicine, Detroit, Michigan; Department of Surgery, Division of Pediatric Surgery, Children's Hospital of Michigan/Wayne State University School of Medicine, Detroit, Michigan; Department of Surgery, Division of Pediatric Surgery, Children's Hospital of Michigan/Wayne State University School of Medicine, Detroit, Michigan

## Abstract

**Introduction:**

Silver sulfadiazine 1% cream was historically the mainstay initial treatment for burn wounds at our pediatric burn center. Over the last several years, we transitioned to using closed dressings of 3% bismuth tribromophenate petrolatum gauze in the initial care of partial thickness burns with the goal of reducing the pain experienced with daily wound cleaning necessitated when using silver sulfadiazine. The gauze adheres to the wound, allows exudate to drain through, and acts as a scaffold for re-epithelialization after which it falls off without traumatizing the healed burn. The purpose of this study is to compare our burn patient outcomes with the initial use of bismuth tribromophenate gauze to those in patients treated with silver sulfadiazine cream.

**Methods:**

A retrospective chart review was conducted of our patients aged 5 years and younger presenting with mixed-depth scald injuries within two time frames: 1) years 2004-2008, during which silver sulfadiazine 1% cream was the standard initial choice for wound care at our institution, and 2) years 2015-2018, when 3% bismuth tribromophenate petrolatum gauze had become standard. Data collected included demographics, burn total body surface area (TBSA), length of hospital stay, need for excision or grafting, time to grafting, and graft size.

**Results:**

Three hundred forty-seven patients were included, 200 of whom were treated with silver sulfadiazine cream and 147 treated with only bismuth tribromophenate gauze. Burn TBSA and rates of skin grafting were similar between the two groups; however, the mean area of the skin graft was significantly smaller (p = 0.027) among the patients in the bismuth tribromophenate group (147 cm^2^) compared to those in the silver sulfadiazine group (336 cm^2^). On the other hand, the time from injury to skin grafting was significantly longer in the bismuth tribromophenate group (24 days vs. 9.9 days, p = 0.002), with a larger proportion of these patients returning for elective grafting in the outpatient setting.

**Conclusions:**

The percentage of patients who required skin grafting in both groups was similar; however, initial treatment with a closed dressing of 3% bismuth tribromophenate petrolatum gauze promotes healing in the zone of stasis, resulting in smaller skin grafts and hence smaller donor sites. The mechanical forces of daily wound cleaning when using silver sulfadiazine cream likely harm those areas at risk, leading to the need for larger skin grafts for coverage. Although time to grafting was longer when using gauze, these patients were in closed dressings with fewer painful changes.

**Applicability of Research to Practice:**

Bismuth tribromophenate petrolatum gauze remains on a wound bed for up to three weeks, whereas silver sulfadiazine cream must be changed regularly resulting in constant disruption of the healing tissue. Fewer dressing changes combined with later skin grafting could allow burn wounds to demarcate and heal more effectively, benefiting both graft and donor sites.

